# The Use of Selective Serotonin Reuptake Inhibitor (SSRI) Antidepressants in the Treatment of Lung Cancer

**DOI:** 10.3390/ijms26104546

**Published:** 2025-05-09

**Authors:** Serap Özkaya Gül, Esra Aydemir

**Affiliations:** Department of Biology, Faculty of Science, Akdeniz University, Antalya TR-07058, Turkey; 202151006001@ogr.akdeniz.edu.tr

**Keywords:** anticancer, antidepressant, cancer, cytotoxicity, drug repurposing, SSRI

## Abstract

Lung cancer is among the most common malignancies globally, is frequently associated with a poor prognosis, and is the second leading cause of cancer-related mortality in both genders. Resistance to treatment, heterogeneity, and invasiveness make lung cancer one of the most challenging tumors to combat. Drug repurposing is considered an advantageous strategy for expediting and economizing drug discovery, as it involves rebranding an existing drug for a new therapeutic use. Since depression is a prevalent psychiatric illness among individuals diagnosed with lung cancer, various selective serotonin reuptake inhibitors (SSRIs) used for the treatment of depression were examined for their possible use in lung cancer treatment as repurposed drugs. Herein, we evaluated the efficacy of SSRIs, both alone and in combination with various anticancer agents, in the treatment of lung cancer along with their mechanisms of action. The innovative approach of repurposing SSRIs offers hope by simplifying the drug discovery process and potentially revealing new therapeutic options for lung cancer. Exploring SSRIs’ effects on lung cancer treatment may unlock unexpected avenues for combating this aggressive disease.

## 1. Introduction

Lung cancer is among the most prevalent cancers, often with poor prognosis worldwide, exhibiting a high morbidity and mortality rate [1]. According to the most recent epidemiological data, it is the second most common cause of cancer-related mortality among both men and women [2]. GLOBOCAN reported around 2.2 million new lung cancer cases (11.4%) and nearly 1.8 million lung cancer deaths (18.0%) in 2020 [3]. The primary risk factors associated with the development of lung cancer include smoking tobacco and using tobacco products, in addition to environmental exposures such as airborne pollutants, biomass fuels, arsenic, radon, industrial carcinogens, and epigenetic alterations [2,4]. The incidence and mortality trends of lung cancer differ substantially among countries, mostly due to varying risk factors, including smoking rates, environmental exposure, and dietary habits [5].

Based on the histology of the cancer cells, lung cancer can be classified into two subtypes: small-cell lung cancer (SCLC) and non-small-cell lung cancer (NSCLC). Non-small-cell lung cancer (NSCLC) is the most prevalent subtype; it accounts for approximately 85% of all lung cancer cases, and SCLC accounts for 15%. NSCLC is highly heterogeneous and has various histological subtypes, including adenocarcinoma (ADC), squamous-cell carcinoma (SCC), and large-cell carcinoma (LCC) [1,6,7]. NSCLC primarily originates in the epithelial lining of the lung and is more challenging to treat with chemotherapy than SCLC. Patients afflicted with this particular form of lung cancer frequently exhibit a poor prognosis, largely attributable to the rapid rate of metastases, elevated relapse rates, delayed detection, and challenges associated with diagnostic modalities. ADC, SCC, and LCC have overall 5-year relative survival rates of 17%, 14%, and 9%, respectively [2]. The most common mutated genes in ADC, the predominant kind of NSCLC, which is more frequently observed in non-smokers and females, include *p53*, *KRAS*, *epidermal growth factor receptor* (*EGFR*), *BRAF*, *PIK3CA*, and *MET* [8]. Squamous-cell carcinoma (SCC) is more prevalent in men and is predominantly linked to smoking behaviors, exhibiting a slower development rate compared to adenocarcinoma (ADC). Squamous-cell carcinoma (SCC) is distinguished by somatic mutations in genes such as *SOX2*, *platelet-derived growth factor receptor A* (*PDGFR-A*), *fibroblast growth factor receptor 1* (*FGFR1*), and *discoidin domain receptor tyrosine kinase 2* (*DDR2*) [9]. SCLC is a highly aggressive, invasive, and lethal form of lung cancer compared to NSCLC, frequently metastasizing to distant organs such as the brain, skeletal system, and lymph nodes and characterized by rapid tumor growth, high vascularity, genomic instability, early metastatic dissemination, almost universal inactivation of TP53 and RB1, and frequent disruption of several characteristic signaling networks. SCLC is associated with more paraneoplastic syndromes than any other cancer and the 5-year survival rate is approximately 7% [6,7,10]. The overall 5-year survival rate for all lung cancer types is low, at 18%, in contrast to other malignancies, such as prostate cancer at 99%, colorectal cancer at 65%, and breast cancer at 90% [2].

The objective of cancer treatment, including lung cancer, is to eradicate or destroy cancerous cells without killing the body’s normal cells. The conventional treatment methods that have been employed most frequently include surgery, radiation, and chemotherapy. These treatment methods can be utilized either individually or in combination with each other [11,12]. Surgery entails the excision of malignant tissue and serves as the principal treatment for the majority of malignancies, especially solid tumors, whereas radiation therapy is the utilization of high-energy X-rays to reduce the size of a tumor. Chemotherapy is the administration of chemicals or drugs whose effects are systemic to kill cancer cells. Chemotherapeutic agents can be classified according to their mechanisms of action, as follows: alkylating agents, antimetabolites, antibiotics, topoisomerase inhibitors, mitotic inhibitors, and corticosteroids [11]. Nonetheless, the efficacy of chemotherapy in lung cancer treatment is often constrained by poor water solubility and half-life, reduced bioavailability due to nonspecific distribution and rapid clearance, and nonspecific targeting, along with significant systemic toxicity and side effects of almost all conventional chemotherapeutic agents [13,14]. Consequently, to restrict off-target toxicity to tolerable levels, the provided chemotherapeutic doses must be constrained, leading to inferior therapeutic efficacy in lung cancer patients. Furthermore, the overexpression of ATP binding cassette subfamily B member 1 (ABCB1), referred to as P-glycoprotein (P-gP), and multidrug resistance protein 1 (MRP1) results in drug resistance in lung cancer cells, significantly contributing to the ineffectiveness of chemotherapy and targeted therapy. Consequently, in clinical applications, additional adjunctive medications are frequently employed to enhance chemotherapy efficacy and mitigate adverse effects, thereby improving treatment outcomes and compliance among patients [14].

Depression is a mood disorder that causes a serious decline in the quality of life of the person, which occurs with continuous feelings of deep sadness and depression, where people’s pleasure and desire to live decrease [15]. Depression causes negative effects not only on mental but also on behavioral and physiological functions. Along with depression, there is an increase in issues such as the person’s loss of self-worth, hopelessness, and the loss of the belief that they can fight against different situations [16]. Although it is seen as an insignificant and negligible disease in the population, depression is among the most common diseases worldwide [17]. Major depression, which is the most common type of depression, occurs approximately twice as often in women as in men. The incidence of major depression may vary between 5 and 12% in men and between 10 and 25% in women [15,18,19].

Psychiatric conditions such as depression and anxiety have been observed to co-occur with chronic diseases, including cancer, cardiovascular disease, and diabetes, in patient populations [20]. Among the diseases that have been associated with depression, cancer has been identified as a condition that frequently arises from the accumulation of a significant number of mutations within the genome [21]. Recent findings indicate that a cancer diagnosis is associated with a higher risk of depression compared to other diseases and contributes to an increased mortality rate in cancer patients [22]. It has been suggested that the onset of major depression due to negative mood is a significant contributing factor to the elevated mortality rate observed in cancer patients [23]. Consequently, depression treatment is often initiated concurrently with chemotherapy in oncology units following a diagnosis of depression [24]. Previous studies have demonstrated that chronic stress, a well-established risk factor for depression, activates the sympathetic nervous system (SNS), which can lead to a decline in the tumor suppressor and anti-angiogenic functions associated with the p53 gene [25]. Consequently, there is an increase in vascularization and an elevated risk of cancer formation after depression caused by chronic stress [25].

Different approaches are adopted in cancer treatment depending on the type and stage of cancer. The efficacy of cancer treatment is contingent upon the judicious selection and adaptation of treatment methods to the patient’s health status [26,27]. The limitations of chemotherapy as a treatment modality are attributed to drug resistance and the development of adverse effects over time. Consequently, there has been a surge of interest in the utilization of FDA-approved drugs as potential alternatives in the field of oncology. This phenomenon, termed “drug repurposing”, involves the identification of alternative uses for therapeutic agents beyond their original indications. This approach facilitates expedited drug development and reduces costs. In the field of oncology, there is considerable promise in the repurposing of drugs that target the central nervous system, including antidepressants and antipsychotic agents [28,29,30].

This review summarizes a general definition of antidepressants used in depression treatment along with current approaches to their classification. In particular, we focused on the anticancer effects of SSRIs, which are frequently used in the depression treatment of cancer patients. SSRIs not only alleviate depressive symptoms but may also play a role in enhancing the overall well-being of patients battling cancer. To provide a broad perspective from approved SSRIs to anticancer drug discovery, the antitumor activity of SSRIs on lung cancer is discussed.

## 2. Classification of Antidepressants

**Figure 1:**
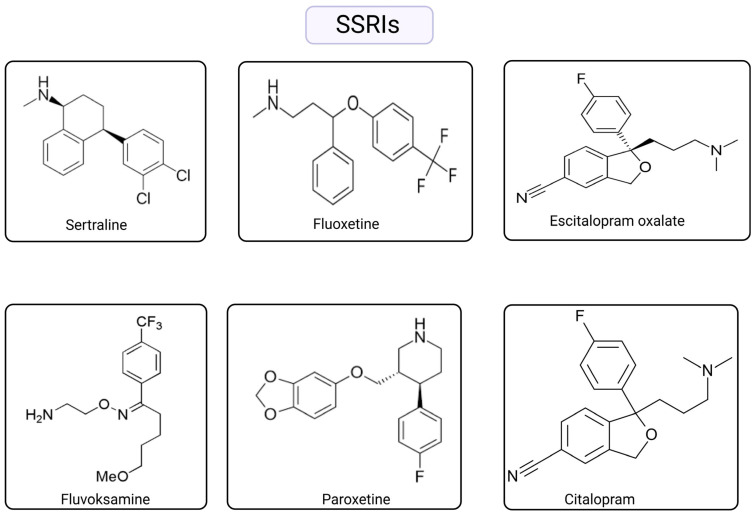
Chemical structures of SSRIs. Created with BioRender (license: https://BioRender.com/e99t72l (accessed on 29 April 2025)).

Antidepressants are drugs that are widely used in mood disorders such as obsessive–compulsive and attention deficit hyperactivity disorder [31], especially in the treatment of major depression and anxiety [32]. The pharmacological processes of these medications involve the alteration of the chemical equilibrium of neurotransmitter molecules in the brain, restoring this balance to normative levels and alleviating symptoms [33]. Furthermore, these medications are classified into two primary categories: first- and second-generation antidepressants. Monoamine oxidase inhibitors (MAO-Is) and tricyclic antidepressants (TCAs) exemplify first-generation antidepressants. Second-generation antidepressants include selective serotonin reuptake inhibitors (SSRIs) (Figure 1), serotonin and noradrenaline reuptake inhibitors (SNRIs), noradrenaline reuptake inhibitors (NARIs), noradrenergic and specific reuptake inhibitors (NASSAs), serotonin antagonist/reuptake inhibitors (SARIs), noradrenaline and dopamine reuptake inhibitors (NDIs), and melatonergic agents [32,34]. However, Alvano and Zieher classified antidepressants into three primary classes based on their mechanism of action as monoaminergic antidepressants, non-monoaminergic antidepressants, and drugs utilized in research and development studies. Monoaminergic antidepressants are further subdivided into four primary subgroups based on their mechanism of action (Table 1) [35].

## 3. Serotonin’s Functions and Its Relationship with Cancer

**Figure 2:**
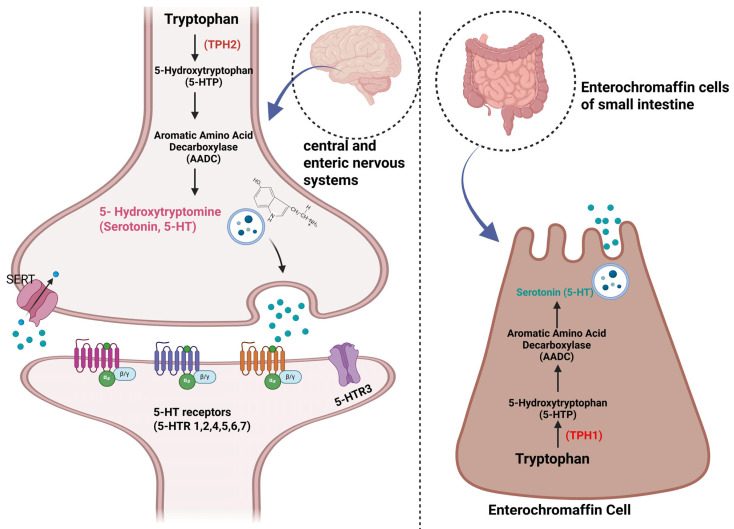
Serotonin (5-HT) biosynthesis by TPH1 and TPH2. Created with BioRender (license: https://BioRender.com/jfal1x0 (accessed on 29 April 2025)).

Serotonin (5-hydroxytryptamine, 5-HT) is a biogenic monoamine that is synthesized from the essential amino acid tryptophan [36]. Serotonin is produced in two steps by the enzymes tryptophan hydroxylase (TPH) and aromatic L-amino acid decarboxylase (AADC) (Figure 2). Due to its inability to traverse the blood–brain barrier, serotonin is produced in two distinct sites in mammals: the brain and the periphery. The synthesis arises through the rate-limiting enzyme existing in two distinct forms, TPH-1 and TPH-2. Approximately 95% of the body’s serotonin is situated in the periphery, predominantly among platelets. Less than 1% of serotonin molecules circulate freely in the bloodstream. Peripheral serotonin production is primarily limited to intestinal enterochromaffin cells and, to a lesser degree, platelets. Platelets function as a significant reservoir for serotonin [37].

The effects of serotonin occur through several specific 5-HT receptors [37]. Since serotonin receptors are expressed in almost all tissues, they have broad physiological roles in the human body. Serotonin synthesized in the brain is involved in neuropsychotic functions such as appetite, sleep, mood regulation, sexual behavior, and pain. Serotonin synthesized peripherally plays important roles in vasoconstriction, angiogenesis, regulation of bone density and osteoporosis, regulation of metabolic functions such as blood glucose levels, and regulation of the gut microbiome [38]. Additionally, serotonin plays important roles in cell growth and differentiation, neuronal development, and associated signaling pathways. Therefore, many pharmaceutical drugs, including antidepressants and antipsychotics, have been developed to target the serotonergic system [37].

**Figure 3:**
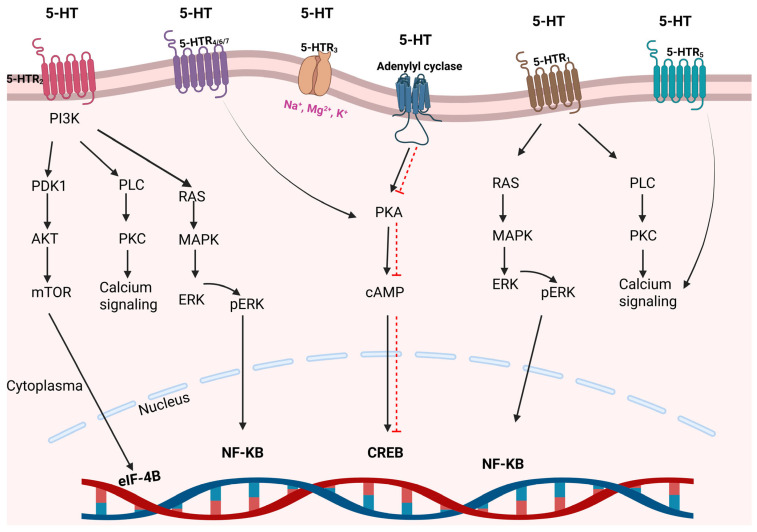
The mechanism of action of 5-HT receptors. Solid black arrows indicate activation, whereas red dotted arrows denote inhibition. Created with BioRender (license: https://BioRender.com/wbxc2r2 (accessed on 29 April 2025)).

The expression of serotonin receptors in various cells across the human body enables them to perform multiple roles. There are at least seven primary receptor families, referred to as 5-HT1 through 5-HT7, that have been identified (Figure 3). There are at least 15 different subtypes of serotonin receptors. The classification of serotonin receptors is carried out based on the structures of the receptors, transduction signals, and pharmacology. Except for 5-HTR3, most of the other 5-HTRs belong to the superfamily of G protein-coupled receptors (GPCR). The 5-HTR3 receptor family functions as a ligand-gated channel that allows the passage of ions such as Na, K, Ca, and Mg upon serotonin binding [39]. The most extensively researched serotonin receptors are of the 5-HTR1, 2, 3, and 4 subtypes. The physiology of the other receptors remains inadequately elucidated. Serotonergic GPCRs consist of a common structure and contain seven transmembrane alpha-helices connected by three extracellular and three intracellular loops. Intracellular C-terminal domain promotes the synthesis of second messengers by engaging with particular families of G proteins such as Gas, Gai/o, and Gaq/11. GPCRs interact with various proteins, including adenyl cyclase (AC), phospholipase C, voltage-gated Ca channels, and K channels [40]. 5-HTR1 receptors can be found in presynaptic serotonergic neurons and postsynaptic non-serotonergic neurons. The 5-HTR1 and 5-HTR5 receptor families are associated with intracellular G-protein Ga i/0 and are also reported to increase ERK phosphorylation via the PI3K/AKT pathway. The 5-HTR2 receptor family is widely expressed in heart and stomach tissues. This receptor family stimulates Ca signaling through phospholipase C activation and exhibits mitogenic activity via the MAPK pathway. The 5-HTR3 and 5-HTR4 receptor family is predominantly expressed in the gastrointestinal system, particularly in the colon and intestines. 5-HTR4, 6, and 7 trigger the PKA/cAMP pathway [39].

The increased expression of 5-HT and its receptors functions as a mitogenic and anti-apoptotic factor and plays an important role in oncogenic progression. Serotonin receptors are expressed in many different types of cancer, including hepatocellular carcinoma, melanoma, and stomach, breast, pancreas, prostate, ovary, bladder, and lung cancers. 5-HTR1A, 1B, and 7 are significantly expressed in cancer, particularly in lung cancer. Moreover, studies indicate that 5-HTR1A and 1D have mitogenic properties in small-cell lung cancer, whereas certain serotonin subtypes, such as 5-HTR3A, 3C, and 5-HTR7, facilitate the proliferation of lung adenocarcinoma, thereby accelerating cancer progression. Serotonin acts as an angiokine in tumor angiogenesis. Platelet stimulation leads to an elevation of serotonin release within the tumor microenvironment. Consequently, through direct interaction with endothelial cells, it can activate the VEGF-mediated PI3K/AKT/mTOR pathway, which are involved in angiogenesis. The influence of serotonin on the tumor vascular system transpires via receptors. 5-HTRs, functioning as tumor suppressors, are expressed less in cancerous tissues. The literature indicates that serotonin receptors may serve as possible targets for inhibiting tumor growth [38].

Clinical studies have identified elevated serum serotonin levels in lung cancer patients with depression. Moreover, it has been demonstrated that the expression of 5-HTR1A-B receptors is elevated in cancer patients with depression relative to those without depression. A negative correlation exists between the expression of 5-HTR1A-B and both the quantity of T cells and the CD8/CD4 T cell ratio. This indicates that serotonin receptors significantly influence the immune system [39]. Serotonin facilitates dose-dependent cellular proliferation in small-cell lung cancer (SCLC) via the 5-HTR1A and 5-HTR1D receptors [37].

## 4. Pharmacokinetics/Pharmacodynamics

SSRIs are considered safe in the treatment of depression due to their specific binding properties. The antidepressant effect of SSRIs is attributable to the inhibition of presynaptic sodium-dependent serotonin transporter (SERT), which results in an increase in serotonin within the synaptic gap. The higher specificity of SSRI group antidepressants for SERT, in comparison to other antidepressants, also prevents the occurrence of antihistaminergic side effects [41]. The half-lives of SSRIs differ based on the specific medication. Sertraline (SRT) and citalopram (CTL) exhibit linear pharmacokinetics, whereas fluoxetine (FLX), fluvoxamine (FVX), and paroxetine (PRX) demonstrate non-linear pharmacokinetics. These differences in pharmacokinetics have the potential to impact dosing strategies and the initiation of therapeutic effects [42].

SSRIs are metabolized through the cytochrome P450 system in the liver. Polymorphisms of enzymes that metabolize and govern the pharmacokinetic behavior of drugs (CYP2D6, CYP2C19, and others) are considered the cause of possible side effects and treatment failures [43]. Each of the SSRIs is metabolized by different cytochrome P450 enzymes. FVX, FLX, and SRT have been shown to exhibit a greater inhibitory effect on P450 enzymes than other antidepressants in the same pharmaceutical group. Consequently, these drugs have the potential to induce a greater number of drug–drug interactions compared to other antidepressants within the SSRIs. All SSRIs have been demonstrated to inhibit CYP2D6, a process that is critical to their pharmacodynamics. Among the SSRIs, fluoxetine has been identified as the most potent inhibitor of CYP2D6, exhibiting the longest half-life [42].

The correlation between pharmacodynamic genes, such as *FKBP5*, *SLC6A4*, *BDNF*, *ABCB1*, *HTR1A*, and *HTR2A*, and clinical outcomes exhibits variability depending on race. In the field of psychiatry and pharmacology, it has been observed that receptors utilized as drug targets for antidepressant medications exhibit a higher degree of evolutionary conservation in comparison to genes that encode for drug metabolism. The prevalence of pharmacokinetic and pharmacodynamic studies is believed to play a pivotal role in enhancing the precision of antidepressant treatments, thereby contributing to the optimization of patient care [43].

## 5. The Anticancer Effects of SSRI Antidepressants in the Treatment of Lung Cancer

**Figure 4:**
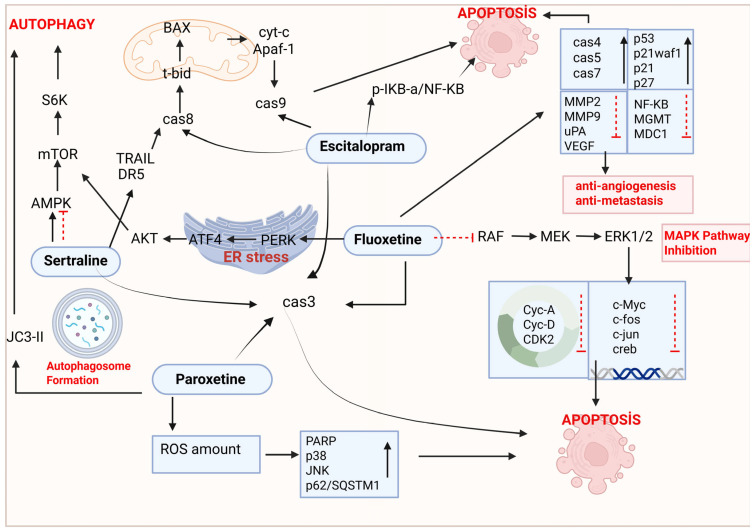
The schematic summary of cellular mechanisms that are involved in the antitumor effects of SSRIs in lung cancer cells. Signaling or drug binding to receptors is symbolized by black arrows. Crossed-out red dashed lines indicate the targets of drug inhibition. Created with BioRender (license: https://BioRender.com/1kiqri9 (accessed on 29 April 2025)).

Antidepressants have been shown to induce distinct death pathways in cancer cells, resulting in cytotoxic effects [44]. The anticancer potential of antidepressants has been thoroughly examined in both in vitro and in vivo models of various cancer types, including breast cancer, lung cancer, gastric cancer, hepatocellular carcinoma, leukemia, and prostate cancer, among others [45,46]. SSRIs, including FLX, escitalopram (ES), SRT, PRX, FVX, and CTL, are frequently used to manage depression in cancer patients. Recent studies indicate that SSRIs exhibit antitumor effects through various pathways [28] (Figure 4). Table 2 lists the effects of FLX, ES, SRT, and PRX among SSRIs in lung cancer and explains their mechanisms. In studies conducted with FVX and CTL, which are among SSRIs, FVX has been shown to be effective in colon [47], breast [48,49], and gynecological [50] cancers; it has been reported that CTL is beneficial in hepatocellular carcinoma [51,52] and neuroblastoma [53] cancers. However, there is no study on lung cancer regarding FVX and CTL.

### 5.1. Fluoxetine

FLX was the first approved SSRI for the treatment of major depressive disorder, bipolar disorder, and panic disorder and is still one of the most prescribed antidepressants worldwide. FLX is commercially available under the brand names Prozac and Sarafem. There are several compelling studies regarding the potential rebranding of FLX for the treatment of various cancer types [42,54,55].

Treatment of A549 cells with 5 μM of FLX for 24 h resulted in a decrease in ERK1/2, mitogen-activated protein kinase, and p-MEK and p-cRaf phosphorylation. FLX-induced inhibition of ERK1/2 phosphorylation was accompanied by a decrease in the phosphorylation of their substrates, c-Myc and CREB, in A549 cells. In FLX-treated A549 cells, while the expression of c-fos, c-jun, cyclin A, and cyclin D1 was decreased, the expression of p21waf1 and p53 genes was increased, and thereby, cell cycle progression was slowed down. In contrast, no changes in the phosphorylation of c-Myc, CREB, or ERK1/2 were found in human skin fibroblasts (HSFs), whose viability was unaffected by FLX treatment [56].

FLX reduced the viability of highly metastatic CL1-5-F4 (NSCLC) cells in a dose-dependent manner (at IC_50_ 40 μM) via the induction of apoptosis and inhibition of NF-κB activation. FLX remarkably decreased the concentration of proteins related to DNA repair, including O6-methylguanine DNA methyltransferase (MGMT), mediator of DNA damage checkpoint 1 (MDC1), and 14-3-3. Since the related levels of metastasis-associated proteins MMP-2, MMP-9, uPA, and VEGF were suppressed by FLX, the metastasis and invasion of CL1-5-F4 were diminished [57].

In consideration of the aforementioned data, Hsua et al. evaluated the in vivo antitumoral activity of FLX on a CL1-5-F4-bearing mice model. The findings revealed that FLX at a dose of 10 mg/kg exhibited a substantial inhibitory effect on tumor growth and size in these established mice models without any general toxicity. FLX was shown to decrease the expression of the cell-proliferative, anti-apoptotic, and invasion-associated proteins Cyclin-D1, survivin, vascular endothelial growth factor (VEGF), matrix metallopeptidase 9 (MMP-9), and urokinase-type plasminogen activator (uPA) on CL-1-5/F4-bearing mice tumor tissue. The expression of AKT or ERK phosphorylation was effectively diminished by FLX treatment in CL-1-5/F4- bearing mice tumor tissue via NF-κB p65 inactivation on ser276. In addition, FLX was also determined to trigger extrinsic/intrinsic apoptotic signaling by activating the apoptosis-associated proteins caspase-3, -8, and -9 in CL-1-5/F4 cells [58].

The in vitro cytotoxic effects of FLX on A549 cells were investigated, and the IC_50_ value for these cells was determined as 15 μmol/L. FLX (20 mg/kg) was shown to inhibit tumor growth, as indicated by weight and size in a xenograft mice model with subcutaneously implanted A549 lung cancer cells. FLX treatment significantly reversed the reduction in serotonin and tryptophan concentrations and suppressed immunity caused by chronic unpredictable mild stress exposure. FLX increased the tryptophan/kynurenine ratio and enhanced cellular immunity. FLX (15 μmol/L) was demonstrated to downregulate the expression of the kynurenine cascade-related genes *tryptophan-2,3-dioxygenase* (*TDO*), *indoleamine-2,3-dioxygenase 1* (*IDO1*), *IDO2,* and *AhR* and upregulate the pro-apoptotic proteins cleaved-caspase 4, 5, and 7, but not cleaved-caspase 1, 3, or 5. It was concluded that FLX induced apoptosis while suppressing migration and colony formation. The antitumoral effects of FLX was attributed to suppressing kynurenine pathway and enhancing cellular immunity [59].

FLX exerted antiproliferative effects on both H460 and A549 NSCLC cell lines by arresting the cell cycle in G0/G1 phase in a concentration-dependent manner. This antiproliferative effect was caused by an increase in the expression of *p21* and *p27* and a decrease in the expression of *CDK2* as well [54]. More importantly, FLX exhibited cytotoxic effects only in cancer cells and not in BEAS-2B cells, which represent normal lung cells. FLX activated the endoplasmic reticulum stress pathway involving PERK, ATF4, and CHOP while concurrently inhibiting the AKT/mTOR pathway in H460 and A549 cells. The transfection of ATF4 small interfering RNA (siRNA) into FLX-treated cells further demonstrated the involvement of endoplasmic reticulum (ER) stress in the inhibition of the AKT/mTOR pathway and the induction of anti-proliferation and autophagy. The results of the study clearly indicated that FLX selectively activated the ATF4-AKT-mTOR signaling pathway, leading to cell cycle arrest and autophagy, hence inhibiting the proliferation of lung cancer cells [54].

In the murine model of experimental lung metastasis conducted by He et al., B16-F10 mouse melanoma cells were injected into the tail vein of C57 mice. Daily treatment with FXT (30 mg/kg) commenced on the third day post-inoculation. It was demonstrated that FXT treatment significantly inhibited the growth of mouse melanoma lung metastasis with no significant changes in body weight. Moreover, the infiltration of immune cells in the lung tumor microenvironment was evaluated after 20 days of FXT treatment. The immune cell population including CD3^+^ cell, interferon-gamma (IFN-γ)^+^ CD8^+^ T-cells, programmed death-1 (PD1)^+^ CD4^+^ T-cells, IFN-γ^+^ CD4^+^ T-cells, nature kill cells, PD1^+^ CD45^+^ T-cells was certainly changed. However, FXT treatment was documented to increase CD8^+^ T-cells, which have a direct function in eradicating cancerous cells by recognizing and eliminating tumor cells. It is noteworthy that the FXT treatment resulted in a substantial augmentation of CD8^+^ T-cells, which play a direct role in the identification and eradication of cancer cells. This study demonstrated the effectiveness of FXT in inhibiting melanoma lung metastasis in preclinical murine models [60].

### 5.2. Sertraline

SRT ((+)-cis-(1S,4S)-N-methy-4-(3,4-dichlorophenyl)-1,2,3,4-tetrahydro-1-naphthylamine) was one of the first SSRIs to be approved by the FDA for medical use and is sold under the brand name Zoloft^®^. SRT has been used effectively to treat major depressive disorder (MDD), panic, generalized and social anxiety, and eating and obsessive–compulsive disorders, as well as premenstrual dysphoric disorder. SRT has two chiral centers with highly selective and potent serotonin reuptake inhibition and is differentiated from other SSRI group antidepressants by its ability to bind to dopamine transporters, improved profile of tolerability, less lethality in cases of overdose, and a reduced risk of dependence [61,62,63]. SRT is mainly metabolized in the liver by cytochrome P450 (CYP) isoforms, including CYP2D6, CYP2C9, CYP2B6, CYP2C19, and CYP3A4. SRT can be demethylated by cytochrome P450 (CYP) enzymes to produce desmethylsertraline, the only active metabolite of SRT. Furthermore, both SRT and desmethylsertraline can be deaminated by CYP3A4, CYP2C19, monoamine oxidase A, and monoamine oxidase B to form alpha-hydroxy sertraline ketone [61]. SRT enhances synaptic transmission by inhibiting the absorption of 5-HT into the presynaptic neuron, thereby increasing the availability of the neurotransmitter for binding to postsynaptic receptors in the synaptic gap. Beyond its role in restoring 5-HT balance within the brain, SRT has also been the subject of studies investigating its anticancer properties in various cancer cell lines [30].

Jiang et al. studied the in vitro antiproliferative activities of SRT in five representative NSCLC cell lines. The cells, with and without *EGFR* mutations, were H522 (*EGFR WT*), A549 (*EGFR WT*; *KRAS* mutation), H1975 (*EGFR T790M* mutation), PC9 (*EGFR 19 bp* deletion in exon 19), and PC9/R which was created by continuously exposing parental erlotinib-sensitive PC9 cells to increasing concentrations of erlotinib for 6 months. With IC_50_ values of 10.50, 11.10, 9.40, 4.40, and 9.60 μM, SRT was found to decrease the viability of H522, A549, H1975, PC9, and PC9/R cells. To understand the mechanism of SRT-induced cytotoxicity, researchers determined the effect of SRT on apoptosis and the cell cycle of NSCLC cells. The target protein poly (ADP-ribose) polymerase (PARP) was not cleaved by caspase-3, and the pan-caspase inhibitor Z-VAD-FMK did not hinder the cell death induced by SRT. According to data obtained, Jiang et al. clearly indicated that SRT did not induce caspase-mediated apoptosis in these cells in vitro. The researchers determined whether autophagy is the mechanism of cell death induced by sertraline in the subsequent stages of the investigation. They accomplished this by Western blot of LC3-II, a pivotal protein in autophagy. In the subsequent stages of the investigation, whether SRT induced autophagy in NSCLC cells was determined by examining the formation of a key biomarker of autophagy, LC3-II, and SRT was found to induce autophagy in a concentration-dependent manner in A549, H522, PC9/R, and H1975 cells. To confirm the induction of autophagy, p62 abundance as a marker of autophagic flux has also been shown to decrease in A549 cells and PC9/R cells. Moreover, SRT was determined to increase the number of autolysosomes and induce autophagic flux in NSCLC cells. SRT cooperatively activates autophagy via targeting the AMPK/mTOR/S6K signaling pathway, which contributes to its cytotoxicity in NSCLC [64].

Zinnah et al. demonstrated that SRT enhanced TRAIL-mediated apoptosis via the downregulation of AMP-activated protein kinase phosphorylation, resulting in the inhibition of autophagic flux, upregulation of *DR5* expression, and activation of the extrinsic pathway of apoptotic caspase cascade by caspase 8 and caspase 3 in the A549, HCC 15, and Calu 3 lung cancer cell lines [65].

### 5.3. Paroxetine

PRX, a well-tolerated phenylpiperidine derivative with a lipophilic amine character, inhibits SERT most potently among the class of SSRIs [66]. PRX, under the brand names Aropax, Brisdelle, Paxil, Paxil CR, Pexeva, Sereupin, and Seroxat, is used for the treatment of major depressive disorder, panic disorder, social phobia, and obsessive–compulsive disorder, as well as generalized anxiety disorder, post-traumatic stress disorder, premenstrual dysphoric disorder, and chronic headache [30,42,66,67].

Aside from its roles in psychiatry as an SSRI, PRX has been shown to exert different biological activities, such as antimicrobial, anti-aging, and anticancer activities [68,69,70,71]. Rosetti et al. evaluated the in vitro cytotoxic activity of PRX on commercially purchased ChaGo-K1 (human NSCLC) and Hude (non-cancerous human fibroblast) cells together with two human NSCLC cell lines obtained and characterized in their own laboratory, which they named CAEP and RAL. Among the lung cancer cells which were exposed to ascending concentrations of PRX for a 24 h period, RAL cells exhibited the greatest sensitivity, with complete inhibition observed at a 5 µM concentration. Furthermore, at concentrations higher than 5 µM, PRX exhibited a cytocidal effect in RAL cells comparable to that of a potent anticancer agent. ChaGo-K1 and CAEP cells were reported to exhibit intermediate sensitivity, with 100% growth suppression at 10 µM, whereas Hude cells were found to be more resistant to the cytotoxic effect of PRX. The mechanism of cell death induced by PRX was demonstrated to be apoptosis using fluorometric imaging of characteristic apoptotic blebs and caspase-3 cleavage analysis [72].

N-methylparoxetine (NMP) is the precursor of PRX. The structural similarity between NMP and PRX suggests they may exhibit analogous physical, chemical, pharmacological, and toxicological properties, indicating potential for comparable clinical applications. The dose-dependent effect of NMP on NSCLC cells, NCI-H1299 and NCI-H1650, was inspected by Wang et al. The IC_50_ for NCI-H1299 and NCI-H1650 cells was found to be 36.97 µM and 45.43 µM, respectively. This anti-proliferative activity was indicated to be specific for NSCLC cells by demonstrating the evidence of its ineffectiveness on a normal lung epithelial cell line (BEAS-2B). NMP not only exhibited cytotoxic effects but also decreased colony formation and inhibited cell division in NSCLC cells. Induction of apoptosis was determined in NSCLC cells by flow cytometry, with an increase in the percentage of Annexin V-positive cells and upregulation of cleaved caspase 3 and cleaved-PARP as well. It has been shown that NMP triggers apoptosis in NSCLC cells through a mitochondria-dependent pathway by upregulating Bax in mitochondrial fractions, leading to outer membrane permeability (MOMP) and an increased release of cytochrome c into the cytoplasm. Since ROS is the main by-product generated by damaged mitochondria, researchers analyzed the amount of intracellular ROS in NSCLC cells and demonstrated that NMP, in a dose-dependent manner, induced the accumulation of ROS. Phosphorylated-p38 and -JNK (active forms), the principal kinases in the MAPK pathway, have been demonstrated to be considerably elevated with NMP therapy due to an increase in ROS levels. Moreover, predicting that the significant increase in the amount of ROS in NMP-treated cells could be caused by the blockade of autophagic flux, the researchers investigated the change in the amount of LC3-II, known as an autophagosome marker. They concluded that NMP treatment in NSCLC cells induced autophagosome accumulation by the upregulation of LC3-II [73].

The potential anti-cancer effects of PRX hydrochloride (Paxil) on NSCLC cells was investigated by Wang et al. The selectivity of Paxil on cancer cells, not on the normal human lung epithelial cell line (BEAS-2B), was declared by IC_50_ values on NCI-H1299 and NCI-H1650 cells of 16.4 and 18.75 µM, respectively. Paxil dose-dependently blocked the colony-forming capability and inhibited the DNA replication of both NCI-H1299 and NCI-H1650 cells. Paxil induced mitochondrial fragmentation at 20 µM, increased the level of ROS in a dose-dependent way in both NCI-H1299 and NCI-H1650 cells, and reduced MMP in NCI-H1299 cells. An elevated level of LC3-II, as an autophagosome marker, after Paxil exposure was shown as the reason for the substantial increase in the number of autophagosomes in NSCLC. The expression level of p62/SQSTM1 protein was evaluated to distinguish between autophagy activation and inhibition of autophagic flux. The elevation of p62 induced by Paxil suggested a blockage in the autophagy system, as p62 is typically degraded via autophagy; however, the upregulation of COX4 elucidated a substantial increase in mitochondrial mass. A dose-dependent upregulation of p-JNK and p-p38, as the major kinases in the MAPK pathway, was illustrated following Paxil induction in NSCLC cells. A dose-dependent upregulation of cytochrome c, Bax, cleaved-caspase 3 and 9, and -PARP levels induced by Paxil in NSCLC cells was demonstrated by Western blot. Apoptosis induction in NSCLC cells was also confirmed via flow cytometry by an increase in the percentage of Annexin V-positive cells [74].

Shao et al. demonstrated that PRX inhibited the viability of H460 and A549 (NSCLC) cells in a concentration-dependent manner at 5, 10, 20, 30, and 40 μM after 24 h of incubation; however, this antiproliferative effect was not selective for NSCLC cells, as PRX also induced cytotoxicity in BEAS-2B cells, which are normal lung epithelial cells [54].

The anticancer effect of PRX (10, 20, and 30 μg/mL) on A549 cells was investigated by Motafeghi et al. In the highest concentrations of PAX, the inhibition rate of A549 cell growth reached 33–43%. At 10 and 30 μg/mL PRX, Ros production was found to be 14.80% and 44.40% in A549 cells, respectively. Moreover, PRX caused 54% lipid peroxidation and decreased GSH reserves by 26% in A549 cells. Since the amount of Ros and lipid peroxidation increased following mitochondrial damage in PRX-treated cells compared to the nontreated control group, the researchers indicated that cell death had occurred due to the induction of oxidative stress. Leakage of lactate dehydrogenase enzyme, condensation and fragmentation of cellular DNA, and induction of apoptosis were detected in A549 cells after 48 h of PRX exposure [75].

### 5.4. Escitalopram

ES, marketed as Cipralex and Lexapro, is a widely prescribed antidepressant belonging to the SSRI class. It is the active optical isomer of citalopram and is utilized in the treatment of serious depression and anxiety disorders. ES is a highly selective, concentration-related inhibitor of the human serotonin transporter and is mainly metabolized in the liver. Compared to the SSRI group of antidepressants, ES and its metabolites are weak inhibitors of CYP2D6 and have been reported to cause no significant inhibition of other CYP1A2, CYP2C9, CYP2C19, and CYP3A4 isoforms [76,77,78]. Previous meta-analyses have indicated that ES is more advantageous than other new-generation antidepressants, and many studies have demonstrated its greater efficacy compared to other antidepressants and placebo. Considering drug interactions, ES is more tolerable than other SSRIs and demonstrates milder or negligible adverse effects [76,77,78].

Similarly to other SSRI antidepressants, ES binds to the orthosteric (primary site) and allosteric binding site of SERT, thereby obstructing the binding of 5HT, the substrate of SERT. SERT inhibition leads to elevated 5HT levels in synaptic clefts in the brain and enhances serotonergic neurotransmission. Among the compounds with allosteric activity with SERT, ES is the most characteristic one. In presynaptic terminals, SERT transports 5-HT from the synaptic cleft back to presynaptic neurons. ES, by binding to the allosteric site, enhances its own binding at the orthosteric site, leading to elevated extracellular 5-HT levels. This results in improved serotonergic neurotransmission, demonstrating impacts on neuronal activity and remodeling, neuroadaptation, and neurogenesis. The binding of ES to the allosteric site may potentially affect the physical interaction between SERT and interacting proteins and thus the modulation of SERT by associated proteins [79]. Liu et al. indicated that the levels of 5HTR1a, 1b, 2a, and 2b receptors were elevated in tumor tissues of lung adenocarcinoma patients. Among these serotonin receptors, especially 5HTR1a and 5HTR1b were demonstrated to always be activated in lung adenocarcinoma patients with depression, and this activation was indicated to be inversely correlated with survival. Particularly, the activation of 5HTR1a is essential for the immunosuppression of lung adenocarcinoma, as it regulates autophagy/STAT3 signaling in patients with depression. Increased expression of 5HTR1a results in a corresponding rise in *BECN1* and *p-STAT3* expression [80].

ES has demonstrated anticancer effects in glioblastoma, gastric, and hepatocellular carcinoma [53,81,82,83,84,85]. Yuan et al. assessed the cytotoxic effects of ES at concentrations of 0.1, 0.2, 0.4, or 0.5 mM on A549 and H460 NSCLC cells, as well as on BEAS-2B human bronchus epithelial cells, through incubation periods of 24 and 48 h. ES has been reported to significantly reduce the viability of NSCLC cells at all concentrations at both incubation times. Sub-G1 proportions and caspase-3 activities were demonstrated to significantly elevate in both A549 and H460 cells treated with 0.1, 0.2, and 0.4 mM ES. Moreover, Bax, tBid, cytochrome c, Apaf-1 and cleaved caspase-9 protein levels were indicated to increase in NSCLC cells that had been treated with 0.2 and 0.4 mM ES for 24 h. Since the expressions of both p-IκB-α and NF-κB (p65-p) proteins were significantly increased in both A549 and H460 cells following treatment with 0.2 and 0.4 mM ES for 24 h, the researchers concluded that ES inhibits the viability of NSCLC cells, resulting in subsequent mitochondria-dependent apoptosis through p-IκB-α/NF-κB (p65-p) signaling [7].

**Table 2:** Summary of findings on anticancer effects of SSRIs in lung cancer.

Drugs	Cell Line(s)	Time	Dose Range	Effective Concentration	Mechanism	Reference
FLX	A549	24, 48 h	1–10 μM	10 μM	Apoptosis	[56]
	CL1-5-F4	48 h	0, 10, 20, 30, 40 μM	40 μM	Apoptosis, anti-angiogenesis, and anti-metastatic effect	[57]
	CL1-5-F4	28 days	dose of 10 mg/kg		Apoptosis, anti-angiogenesis, and anti-metastatic effect	[58]
	A549 and mouse model	72 h (in vitro) and 7 days (in vivo)	15 μmol/L and 20 mg/kg (mice)	15 μmol/L	Apoptosis	[59]
	H460 and A549	24 h	0, 5, 10, 20, 30, and 40 μM	20 μM	Autophagy	[54]
SRT	H522, A549, H1975, PC9, and PC9/R	72 h	0, 5 and 10 µM	10.50, 11.10, 9.40, 4.40, and 9.60 μM	Autophagy	[64]
	A549 and HCC 15 vs. Calu 3	18 h	0, 2.5, 5 and 10 µM	10 µM	Extrinsic pathway of the apoptosis	[65]
PRX	ChaGo-K1 and Hude	24 h	5, 10, 50, 100 µM	5 and 10 µM	Apoptotic effect	[72]
	NCI-H1299 and NCI-H1650	24 h	0, 10, 20, 3 40, 50, 60, 70 µM	36.97 µM and 45.43 µM	Mitochondria-dependent apoptosis	[73]
	H460 and A549	24 h	0, 5, 10, 20, 30, and 40 μM	20 μM	Antiproliferative effect	[54]
	A549	48 h	10, 20, and 30 μg/mL	30 μg/mL	Increased amount of ROS, mitochondria-dependent apoptosis	[75]
ES	A549 and H460	24 h	0.1, 0.2, 0.4, or 0.5 mM	0.2 and 0.4 mM	Mitochondria-dependent apoptosis	[7]

When studies evaluating the anticancer effects of SSRIs on lung cancer are compared, it is evident that all SSRIs activate the apoptosis mechanism. Studies conducted with FLX, ES, and SRT clearly show that the anticancer mechanism of these agents is apoptotic. However, when SSRIs are evaluated in terms of the autophagy mechanism, it is observed that FLX and SRT are more significant in this mechanism [7,54,56,57,58,59,60,64,65]. There are very limited studies in the literature regarding the effectiveness of SSRIs in lung cancer in terms of ROS production, anti-angiogenesis, and anti-metastatic activities. Studies have demonstrated that PRX elevates ROS levels, while FLX demonstrates anti-angiogenic and anti-metastatic properties in lung cancer cells [54,56,57,58,59,60,72,73,75]. The literature reveals that SSRIs are effective in lung cancer through similar mechanisms, but the effect profiles of each drug can differ on a mechanistic basis (Table 3). This discrepancy suggests that further research is necessary to fully understand how different SSRIs may uniquely influence the viability of lung cancer cells. Investigating these variations could lead to more tailored therapeutic strategies for patients with lung cancer, ultimately improving treatment outcomes.

## 6. The Synergistic Effects of SSRIs in Lung Cancer Treatment

Depression is recognized as a primary contributor to elevated mortality rates in cancer patients. In oncology units, people receiving chemotherapy also receive depression treatments. SSRIs are more frequently used in combination therapy due to their reduced adverse effects relative to other kinds of antidepressants. Antidepressants produce a synergistic effect through pharmacological interactions when administered concurrently with chemotherapy drugs. This concurrent utilization is not only an interaction at the pharmacological level but also a creation of molecular synergism. SSRIs enhance the cytotoxic effects of chemotherapeutic agents by activating apoptotic (e.g., caspase-3, BAX/Bcl-2 balance) and oxidative stress pathways (e.g., ROS production) and cellular stress responses (e.g., PERK/ATF4 pathways) as well. Furthermore, SSRIs may suppress proliferative signal-ing, such as mTOR/AMPK and ERK1/2, thereby overcoming mechanisms of resistance to chemotherapeutic drugs. In particular, the enhancement of apoptosis via TRAIL/DR5 by ES or the induction of autophagy via mTOR inhibition by sertraline has the potential to increase the efficacy of chemotherapy in specific combinations. Furthermore, it enhances sensitivity to chemotherapeutic drugs in cancer cells exhibiting multidrug resistance, thus helping in overcoming this resistance. Beyond their cytotoxic and synergistic effects, the chemosensitive features of antidepressants shown in resistant cancer cells offer promise that the efficacy of cancer therapies may be improved by their concurrent use [44].

A recent study indicates that the synergistic application of SSRIs alongside standard anticancer agents enhances the efficacy of tumor cell therapy. A substantial body of research has demonstrated the anticancer properties of the SSRI group of antidepressants exhibit a synergistic effect with anticancer agents in numerous types of cancer, including lung, breast, glioblastoma, and colon [60,64,86,87,88,89,90,91,92]. While numerous cancer types have been documented for the combined effects of SSRIs and anticancer drugs, research specifically on lung cancer remains limited. Table 4 presents studies investigating the impact of SSRIs in combination therapy for lung cancer.

A study examining the possible drug–drug interaction between PRX and dacomitinib indicated PRX as an appropriate candidate for phase I trials. While PRX significantly inhibits CYP2D6-mediated metabolism of dacomitinib, this metabolic pathway is not the primary elimination mechanism of dacomitinib. Dacomitinib is subjected to glutathione conjugation and CYP3A4-mediated metabolism to produce several oxidative metabolites. Further experience with multiple dosing of both PRX and dacomitinib in NSCLC will help to establish whether the interaction between these drugs is effective in cancer treatment [93]. The study on the SRT/Erlotinib combination revealed a synergistic impact by promoting autophagy in non-small-cell lung cancer cells, evidenced by the increase in autophagic markers (LC3) and the development of autophagic lysosomes. Although EGFR mutant lung cancer cells initially respond to TKIs, treatment efficacy gradually weakens as tumor cells rapidly develop resistance to these inhibitors. Erlotinib binds to the ATP binding site of EGFR, dephosphorylates EGFR, and disrupts the interaction between EGFR and the autophagy protein Beclin 1. Stimulation of the autophagy pathway increased the efficacy of erlotinib by disrupting survival mechanisms in cancer cells [64]. Another study with sertraline and radiotherapy combination revealed that the combination reduced the growth of NSCLC, together with the cloning capability and the self-renewal potential of stem cells. Furthermore, an in vivo investigation demonstrated that the combination diminished NSCLC tumor development and altered the tumor microenvironment via cytokines linked to natural killer cells [94]. Zinnah et al. showed that the combination of SRT and TRAIL increased TRAIL sensitivity in TRAIL-resistant lung cancer cells by suppressing AMP-activated protein kinase (AMPK) phosphorylation. This resulted in an inhibition of the autophagic flux, increased DR5 receptor expression, and activation of the extrinsic apoptotic pathway via caspase-8. Consequently, apoptosis was found to be elevated in the A549, HCC-15, and Calu-3 cell lines [65]. The examination of the fluoxetine/paclitaxel combination’s efficiency on lung cells revealed that this combination is cytotoxic to cancer cells while being safe for healthy epithelial cells at lower dosages. Furthermore, palmitoyl-protein thioesterase 1 (PPT1) is observed to potentially play a significant part in the mechanism of action of these combinations [62].

There are important differences between each SSRI that may influence the choice of treatment. Due to its long half-life and potent cytochrome P450 (CYP450)-inhibitory effects, FLX should be used with caution in cancer patients receiving chemotherapy to avoid the risk of drug interactions with anticancer agents metabolized through the CYP450 system. Similarly, paroxetine has marked P450 inhibitory effects and anticholinergic effects that should be carefully monitored. SRT, CTL, and ES have the fewest drug–drug interactions and are the best first-line treatment option. These drugs tend to be well tolerated, but caution is needed at higher doses due to QTc prolongation and the antiplatelet effects of SSRIs [95]. Possible pharmacokinetic and pharmacodynamic interactions should also be considered in the concomitant use of SSRIs with chemotherapy drugs. FLX and PRX, in particular, are potent inhibitors of the cytochrome P450 (CYP450) enzyme family. The concomitant use of SSRIs with chemotherapeutic agents (e.g., tamoxifen, vinorelbine, etoposide), especially those metabolized via CYP2D6 and CYP3A4 isoenzymes, may result in increased plasma levels of these drugs and thus increased risk of toxicity. Certain SSRIs, which are strong inhibitors of CYP2D6, may cause adverse effects when used in combination with anticancer drugs such as tamoxifen. To avoid such adverse interactions, choosing SSRIs with a low CYP450 inhibition profile, such as SRT or ES, may be a safer strategy [96]. It is known that most antineoplastic drugs metabolized by CYP3A4 and certain antidepressants demonstrate inhibitory effects on CYP3A4. Consequently, the interactions between drugs and intermediate metabolites in combinations of anticancer agents with SSRIs must be thoroughly evaluated and studied in clinical settings [97].

**Table 4:** Anticancer effects of SSRI combinations on lung cancer.

Drugs	Cancer	Time	Doses	Mechanism	Reference
PRX/Dakomitinib combination	NSLCL	10 days	45 mg	Potential drug–drug interaction and cytotoxic effect	[93]
SRT/Erlotinib combination	PC9, PC9/R, A549, H522 and H1975	24 h	5.10 vs. 20 μM	Exhibits a synergistic effect by promoting autophagy through the increase in autophagic markers (LC3) and the development of autophagic lysosomes	[64]
SRT/Radiation combination	A549, H1299, H125, H520, H1975, HCC15, and Calu-6	24 h	10 µM SRT, 6 Gy	Reduces NSCLC tumor growth and alters the tumor microenvironment through cytokines linked to natural killer cells	[94]
SRT/TRAIL combination	A549 and Calu-3 vs. HCC-15	18 h	SRT (0, 2,5, and 5 vs. 10 µM) TRAIL (100 ng/mL)	AMPK inhibits the autophagic flux in TRAIL-resistant cells via TRAIL-mediated apoptosis and extrinsic apoptosis in A549, HCC-15, and Calu-3 cells.	[65]
FLX/Paclitaxel combination	MRC-5	48 h	6.12 µM and 2.61 nM	Palmitoyl-protein thioesterase 1 (PPT1)	[29]
PRX/Amitriptyline combination	A549	48 h	10, 20, and 30 μg/mL	Inhibits the growth of cancer cells by inducing apoptosis and LDH leakage and inducing oxidative stress	[75]
(Metformin/FLX), (Efavirenz/FLX), and (Metformin/Efavirenz/FLX) combination	A549	24 h	Metformin (5 mM), Efavirenz (1.5 μM), FLX (0.9 μM)	Increased cellular ROS levels	[98]
FLX/Gefitinib or FLX/Erlotinib			0.1 to 100 μM	Pre-clinical drug–drug interaction (DDI)	[99]
PRX/Cisplatin combination	H1299	24 h	0, 10, 15, and 20 μM	Increased ROS, JNK, and p38 activation and intrinsic apoptosis	[74]
ES/Etoposide	A549, H1299, A54990E	24 h	ES (5, 10, 25, 50, 100, 200, 250, 400, and 500 μg/mL) and Etoposide (5, 25, 50, 100, 250, and 500 ng/mL)	Triggers cytotoxic and apoptotic activity and reduces resistance to etoposide by reducing the amount of P-gP	[100]

## 7. Conclusions

Drug repurposing provides benefits including determining new indications for existing drugs, the utilization of risk-free compounds, reduced development timelines, and potentially diminished overall development costs. SSRIs alone and in combination with anticancer agents cause cytotoxic effects on lung cancer cells. This situation holds promise for increasing the effectiveness of cancer treatment in patients who are currently undergoing lung cancer treatment concurrently with SSRIs. Given that SSRIs modify drug resistance in cancer cells and enhance their sensitivity to anticancer drugs, they may serve as an alternative for patients exhibiting resistance to chemotherapy. Moreover, it is essential to recognize that the administration of SSRIs at acceptable dosages, in conjunction with suitable drugs, will significantly influence the efficacy of the lung cancer treatment.

## Figures and Tables

**Table 1:** Classification of antidepressants according to their mechanism of action. Adapted from Alvano and Zieher (2020) [35].

Category	Drug Class	Mechanism	Generic Names
First generation	Tricyclic antidepressants (TCAs)	İnhibit reuptake of norepinephrine and serotonin into presynaptic terminals	Amiltriptyline, Clomipmine, Desipramine, Doxepin, İmipramine, Nortriptyline, Amoxapine, Protriptyline, Trimipramine
	Monoamine oxidase inhibitors (MAOIs)	Competitively inhibit monoamine oxidase; agents in the class differ in their reversibility and their activity against MAOOa and MAOb	Tranylcypromine, Phenelzine, Selegiline, Isocarboxazid
Second generation	Selective serotonin reuptake inhibitors (SSRIs)	Selectively inhibit the reuptake of serotonin (5-HT) at the presynaptic neuronal membrane	Fluoxetine, Fluvoxamine, Paroxetine, Sertraline, Citalopram, Escitalopram
	Selective norepinephrine reuptake inhibitors	İnhibit reuptake of both serotonin and norepinephrine; weakly inhibit dopamine reuptake	Venlafaxine, Mirtazapine, Duloxetine
	Serotonin antagonist and reuptake inhibitors (SARIs)	5-HT2 receptor antagonists	Nefazodone
	Dopamine reuptake inhibitors	İnhibit dopamine reuptake with some effect on norepinephrine	Bupropion

**Table 3:** Summary of findings on anticancer effects of SSRIs in lung cancer.

Drugs	Apoptosis	Autophagy	ROS Production	Anti-Angiogenesis/Anti-Metastasis
FLX	+	+	−	+
ES	+	−	−	−
SRT	+	+	−	−
PRX	+	+	+	−
FVX	+	−	−	−

## Data Availability

Not applicable.

## Abbreviations

The following abbreviations are used in this manuscript:

MDPI	Multidisciplinary Digital Publishing Institute
DOAJ	Directory of open access journals
TLA	Three letter acronym
LD	Linear dichroism
